# Christmas

**Published:** 1879-01

**Authors:** 


### CHRISTMAS.

Well, the whirligig of time has brought us to another Christmas. Gradually, are our little ones outgrowing the beautiful legend of good old Santa Claus, until but two little innocents at this time remain, in our own immediate household, young enough to take comfort and delight in his coming. The circumstance reminds us of approaching age and of the time when Santa Claus' coming will have no further charms for us; when the children shall have outgrown him, leaving us to settle down to the stern realities of life. So, let us have a good time while we can, and join in the festivities of the holidays with the little folks, so adding to their pleasure as well as our own.

Nothing can add so much to our happiness as to witness the delight of the dear little children, as they bound into mamma's and papa's room early on Christmas morning, to tell of the wonderful things that Santa Claus brought to them during the night. How their innocent eyes sparkle as each pretty plaything is held up for inspection, and how they scamper to the parlor to bring other trophies from Santa Claus' inexhaustable bag, as you join in the exclamations of surprise at his bounty.

Bless the dear little children; how we wish it were possible for Santa Claus to remember them all, never missing one, as he drives his mysterious equipage over our house-tops.

Will not the grown up folks help remind Santa Claus to stop at the houses of poor little children, that he might somehow forget? We hope so, and that every little child in the land may have some reminder of his goodness and loving kindness.

